# Mapping health services for adults with cerebral palsy in Ireland: a pilot study

**DOI:** 10.12688/hrbopenres.13609.2

**Published:** 2022-12-15

**Authors:** Manjula Manikandan, Shalini Jagdeo, Fiona Weldon, Sarah Harrington, Rory O'Sullivan, Jennifer Fortune, Claire Kerr, Jennifer M Ryan

**Affiliations:** 1 Department of Public Health and Epidemiology, Royal College of Surgeons in Ireland University of Medicine and Health Sciences, Dublin, Ireland; 2 Public and Patient Involvement contributor, Meath, Ireland; 3 Strategies for Change Co-ordinator, Independent Living Movement Ireland, Dublin, Ireland; 4 Public and Patient Involvement contributor, Cork, Ireland; 5 Adult Continuing Education, University College Cork, Cork, Ireland; 6 Central Remedial Clinic, Dublin, Ireland; 7 School of Nursing and Midwifery, Queen's University Belfast, Belfast, UK; 8 College of Health, Medicine and Life Sciences, Brunel University, London, UK

**Keywords:** Cerebral palsy, health services, adults, mapping, survey.

## Abstract

**Background**: Cerebral palsy (CP) is a common cause of physical disability in childhood. The majority of children with CP survive to adulthood. Once discharged from children’s services, adults with CP find it challenging to navigate health services. The aim of this study was to pilot and refine a methodology to map services for adults with CP in Ireland.

**Methods:** We used a multi-informant mapping methodology consisting of: 1. Defining health services; 2. Identifying informants; 3. Designing a survey; 4. Collecting data; 5. Data checking and analysis. We collected data on services from service users and service providers using an online survey. We verified data against information available online and by asking organisations to provide details about the service.

**Results:** Fifteen service users and nine service providers completed the online survey. Data on 265 unique services at 32 organisations were provided. The most commonly provided services were physiotherapy (12%) and occupational therapy (11%). We confirmed the name of 89 services (34%) against online information. We received further details from eight organisations about 27 services. Specifically, we received details about the organisation name for 27 of the 265 services (10%), service name for 25 services (9%), service type for 25 services (9%), a website for 19 services (7%), and data on eligibility criteria and types of supports provided for between 25 or 26 services (9% or 10%).

**Conclusion**: This pilot study highlighted the complexity of mapping services for adults with CP in Ireland. We recommend that an alternative methodology should be used to map services for adults with CP in Ireland.

## Introduction

Cerebral palsy (CP) describes “a group of permanent disorders of the development of movement and posture, causing activity limitation, that are attributed to non-progressive disturbances that occurred in the developing fetal or infant brain” ^1^. People with CP may experience associated impairments and secondary conditions such as epilepsy, sensory impairment and intellectual disability ^1^. The prevalence of CP in Ireland is approximately 2 per 1,000 live births ^2– 4^, which, based on the number of births in Ireland per year ^5, 6^, suggests there are at least 2,700 children with CP aged 0–18 years living in Ireland. At least 90% of children with CP survive to adulthood ^7^. The period prevalence of CP among adults reported recently from the Northern Ireland CP Register (NICPR) was 2.38 per 1000 population born between 1981–2001 ^7^. Based on the NICPR report ^7^ and the population of adults in the Republic of Ireland ^5^ we estimate there are approximately 6,800 adults living with CP in Ireland.

Many adults with CP experience complications such as fatigue, pain and decline in mobility ^8^. They are also more likely to experience chronic conditions such as cardiovascular disease, arthritis, depression and anxiety than adults without CP ^4– 6^. As such, people with CP require services and supports that optimise health and participation in everyday activities across the lifespan. These include therapy services, respite services, personal assistance and medical services. However, adults with CP typically report that there are a lack of appropriate services to meet their needs ^9^. People with CP report that the period when they are discharged from children’s services is particularly challenging because they no longer have access to coordinated care and have difficulty identifying and navigating services to meet their needs ^10– 12^. This is compounded by the fact they receive little information about the process of being transferred from children’s to adults’ services ^13^.

National strategies in Ireland, such as the “National Policy and Strategy for the Provision of Neuro-Rehabilitation Services in Ireland” and the “Integrated Care Programme for Children”, emphasise the need for appropriate planning to support the transfer from children’s to adults’ services ^14, 15^. Collating data on services available to adults with CP in Ireland may support health professionals to better plan transfer and may inform development of coordinated, evidence-based services for adults with CP in accordance with the National Policy and Strategy for the Provision of Neuro-Rehabilitation Services ^14^. Further, people with CP and their families in Ireland report that having a map of services and supports available to adults with CP would help them to navigate services in adulthood and improve the experience of being transferred to adults’ services ^16^.

Recently, a multi-informant mapping methodology was used to efficiently collate and communicate data on health services for adults with attention-deficit/hyperactivity disorder (ADHD) in the United Kingdom ^17, 18^. While this method may be used to map services for adults with CP in Ireland, it needs to be piloted and refined to ensure that accurate data is collected. Therefore, the aim of this study was to pilot and refine a methodology to map services for adults with CP in Ireland.

## Methods

### Ethics

Ethical approval was obtained from the RCSI University of Medicine and Health Sciences Research Ethics Committee (Ref: REC202105004). All data were anonymous. Completion of the survey indicated consent.

We piloted a methodology that was used to map services for adults with ADHD in the UK.

The methodology consisted of seven steps: 1. Defining health services; 2. Identifying informants; 3. Designing a survey; 4. Collecting data; 5. Data checking and analysis; 6. Communicating findings; and 7. Updating findings ^17^. We report data relating to steps 1–5 and share plans for communicating findings.

### Public and patient involvement

Two adults with CP were part of the research team and contributed to all stages of the study.

### Health services definition

We broadly defined health services as “ *any health service for people with CP aged 18 years and above located in Ireland*”. Through discussion with the research team, including adults with CP, we specified that *“we consider health services are those that are first and foremost undertaken to have direct effect on people’s health. These extend from health promotion and disease prevention, through curative services, to long-term care and rehabilitation*” ^19^. Adults with CP thought it would be more useful to provide examples of what we did and did not consider to be a health service. We agreed that health services may include any medical service (e.g., rehabilitation specialist, paediatrician), therapy service (e.g., physiotherapist, occupational therapist), social work service, diagnostic service, assistive technology service, support service (e.g., personal assistance, respite), disability service, charity, or support group that treats or supports adults with CP. This description was provided when collecting data on services using a survey. We also included exercise groups, gyms or swimming pools that are accessible to people with disability in the examples of services because they support health promotion and adults with CP on the research team thought information on these facilities is important to collate.

### Informants

We identified informants as adults with CP, people who support adults with CP (e.g., family members, friends, personal assistants), and people who provide services to people with CP (e.g., clinicians, health and social care professionals, managers). We categorised informants as service users (i.e., adults with CP and support people) and service providers. We included service providers working in paediatric and adult settings.

### Data collection

***Survey.*** While developing the survey, we focused on collecting only necessary data and keeping the number of questions to a minimum. We developed a survey to collect the following: respondent characteristics including their perceived primary role (e.g. adult with CP, physiotherapist), secondary role (if any), geographical location; service details including name of organisation, name of service (e.g. physiotherapy, assistive technology), location(s) of service, and website. We also asked informants if they knew of someone aged 18 years or older who received assessment, treatment or support at the service for their CP, if the service was delivered through public or private healthcare or both, and if the respondent believed the service had expertise in treating or supporting adults with CP. The survey was hosted on onlinesurveys.ac.uk. The survey was piloted by two adults with CP, a paediatrician and a physiotherapist who worked with people with CP. During piloting, we identified that when a single question asking for the name of the service was included, some informants provided an organisation name only and some provided detail about the type of service. We therefore separated this question into two questions that asked for (1) name of organisation and (2) name of service (e.g., physiotherapy).

The survey was distributed to 24 service users and 35 service providers through the research team’s network. As the aim of this study was to pilot and refine the methodology, we limited the number of people who received the survey so that we could refine the methodology based on their responses before distributing it more widely. After participants completed the survey, they were asked to provide anonymous feedback on the survey via a free text box or to provide their contact details so the research team could contact them to obtain feedback. The survey opened on 28 ^th^ July 2021 and closed on 3 ^rd^ September 2021.

### Data checking and analysis

***Data checking.*** During data checking, we aimed to verify the information provided by informants. Survey responses were downloaded to Excel. Each informant and service was given a unique ID number. A service was deemed duplicated if two or more people provided an identical organisation name, service name, and service location (i.e., all three pieces of information had to be identical). Where an organisation provided just one service nationwide, we deemed this service to be duplicated if two or more people provided the same organisation name only. One informant provided information on physiotherapy, occupational therapy, speech and language therapy, social work, counselling, home help and personal assistance provided by the Health Service Executive (HSE) disability services and stated each service was provided “county wide”. We therefore treated each service in each county as one service (e.g., physiotherapy provided in county Mayo was one service). However, where a service was provided nationwide, we treated it as one service only.

We checked data for each unique service by (1) searching online and (2) sending a survey to the providing organisation. We checked the exact service name and location provided by the informant. If an organisation name but no service name was provided, we were unable to verify any information for the service unless the organisation provided only one nationwide service. Similarly, if a service name but no organisation name was provided we were unable to verify any information for the service.

Two researchers independently searched for information online, compared their findings and resolved any disagreements through discussion. They firstly searched the website provided by the informant. They additionally entered search terms relating to the organisation, service and location into a search engine. If we could not find the specific service name online, we could not verify the service. Information obtained online was: organisation name; service name; website or webpage associated with the service; location of the service; if the service was a specialist CP service (determined if the online information specifically stated that the service was provided to adults with CP); and eligibility criteria. The information obtained in relation to eligibility criteria were: (1) lower age boundary; (2) upper age boundary; (3) if adults without intellectual disability were eligible; (4) any other eligibility criteria. We also sought to identify if the service provided any of the following: transitional support; review by a specialist multidisciplinary team; an annual review with a healthcare professional with expertise in neurodisability; a review by a professional with expertise in vocational skills; review by a professional with expertise in independent living; referral to a professional with expertise in vocational skills; referral to a professional with expertise in independent living; assessment by speech and language therapy services. These were selected based on the Quality Standard covering care and support for adults with CP developed by the National Institute for Health and Care Excellence (NICE) ^20^. The Quality Standard describes ‘high-quality care in priority areas for improvement’ for adults with CP ^20^.

An email containing a link to an online survey was sent to organisations to verify their service(s). Organisations could also request a paper version of the survey, a soft copy of the survey to complete over email or could complete it over the phone. If there was no response to the email within a week or an email address could not be located for the organisation, the organisation was contacted by phone. If there was no response following an email and/or phone call, the organisation was sent a second email with a link to the survey.

***Analysis.*** Data were analysed using Stata version 15.1. Descriptive statistics (frequencies and percentages) were used to describe informant characteristics. To preserve anonymity, we combined the counties that informants lived or worked in to create larger categories. Descriptive statistics (frequencies, percentages, medians, interquartile range [IQR], minimum and maximum) were used to report data relating to organisations and services.

## Results

### Description of informants

Twenty-four people completed the survey. Of these, 15 (63%) identified as service users and nine (37%) identified as service providers. Of the 15 service users, nine were adults with CP and six were parents, guardians or other family members of adults with CP. Four service users provided a secondary role, which were a researcher/academic, personal assistant and medical or allied health professionals. Five service users lived in Dublin (33%), five lived in Cork (33%) and the remaining lived in three other counties. 

Of the nine service providers, three were physiotherapists, three were occupational therapists, two were paediatricians and one was a social worker. Five service providers worked in Dublin (56%). The remaining four reported working in the south (i.e., Munster) and east of Ireland (i.e. Leinster excluding Dublin). Service providers stated they worked in the voluntary sector only, HSE only, private sector only, or HSE and voluntary sector.

### Description of services

Four informants provided no data on an organisation and/or service and were excluded. One informant provided a service name but not an organisation name. Six informants provided eight responses that included an organisation name but not a service name. After removing these nine responses, data from 18 informants were included in the analysis. The median (IQR) number of organisations reported per informant was 2 (3) (minimum-maximum 1–11). The median (IQR) number of services reported per informant was 3 (4) (minimum-maximum 1–211). Thirteen responses were duplicates. We describe the 265 unique services at 32 organisations reported by informants. Where duplicate responses were provided by informants, they sometimes provided conflicting information about the service. For example, one informant might state the service has expertise in treating or supporting adults with CP while a second informant might state the service does not have expertise or they do not know if the service has expertise. For services where conflicting information was provided by informants, we counted the service as “yes” (e.g., has expertise) if at least one person responded yes to the question. 

The organisations and the type of service(s) they provide, as identified by informants, are described in Table 1. The counties in which services were located are provided in Table 2. Location was not provided for three services. Eight services (3%) were described as nationwide. Forty-three services (16%) were located in Dublin, 37 (14%) in the south-east region, and 36% (13%) in the border region.

**Table 1.** Organisations provided by informants.

Organisation	Unique services at organisation, n (%)	Type(s) of service provided	Private/Public
Health Service Executive	187 (70.6%)	Physiotherapy Occupational therapy Home help/personal care Personal assistance Speech and Language therapy Social work Psychiatry/counselling Assistive technology and Special Seating Advocacy service	Public
Enable Ireland	19 (7.2%)	Physiotherapy Occupational therapy Speech and Language therapy Day service Assistive technology and Special Seating Hydrotherapy Training and employment service Adult service/service for adults with physical disabilities Respite	Both
Irish Wheelchair Association	13 (4.9%)	Home help/personal care Personal assistance Training and Employment Adult service/service for adults with physical disabilities Assisted/independent living Sports groups/clubs Strengthening or exercise facilities Resource centre Driving school	Public ^a^
Central Remedial Clinic	12 (4.5%)	Physiotherapy Occupational therapy Social work Assistive technology and Special Seating Hydrotherapy Orthotics and Prosthetics Medical neuro team Feeding eating and drinking Hand function Orthopaedics	Public
Barefoot Physiotherapy	3 (1.1%)	Physiotherapy Strengthening and exercise facilities Relaxation, meditation and massage therapy	Private
Celbridge Medical Centre	3 (1.1%)	Physiotherapy Hydrotherapy Sports groups or clubs	Private
Apos ltd	2 (0.8%)	Orthotics and Prosthetics	Public
The Dublin Neurological Institute	2 (0.8%)	Neurology Relaxation, meditation and massage therapy	Public
Abode	1 (0.4%)	Respite	Unknown
Barrog Healthcare	1 (0.4%)	Personal assistance	Private
Blackrock Hall	1 (0.4%)	Physiotherapy	Public
Brothers of Charity	1 (0.4%)	Day service	Public
Caring and Sharing Association	1 (0.4%)	Social club	Unknown
Centre for Independent Living *	1 (0.4%)	Advocacy service	Public
Cheeverstown	1 (0.4%)	Day service	Public
Cheshire Home	1 (0.4%)	Residential house	Unknown
COPE Foundation	1 (0.4%)	Adult service/ service for adults with physical disabilities	Public
Curam	1 (0.4%)	Psychiatry/counselling	Public
Disability Federation of Ireland	1 (0.4%)	Wheelchair accessible beaches	Unknown
Dublin Hydrotherapy	1 (0.4%)	Hydrotherapy	Private
Finglas Sports Centre	1 (0.4%)	Strengthening and exercise facilities	Private
Galway Speeders	1 (0.4%)	Sports group or clubs	Public
Independent Living Movement Ireland	1 (0.4%)	Assisted/ independent living	Public
Irish Pilgrimage Trust	1 (0.4%)	Respite	Unknown
Killeen *	1 (0.4%)	Day service	Public
Kiltipper Woods physiotherapy and hydrotherapy	1 (0.4%)	Hydrotherapy	Private
Mater Hospital pain clinic	1 (0.4%)	Pain clinic	Public
National Advocacy Service	1 (0.4%)	Advocacy service	Public
New Horizons	1 (0.4%)	Day service	Public
St Joseph’s Foundation	1 (0.4%)	Adult service/ service for adults with physical disabilities	Public
WALK	1 (0.4%)	Training and employment service	Unknown
Waterford Intellectual Disability Association	1 (0.4%)	Day service	Public

*Unable to confirm organisation name against online information

^a^Respondents also reported unknown for some services

**Table 2.** Nationwide regions and counties in which services were located.

Region ^a^ and counties	Number of unique services (n)	Percentage of total number of services (n=265) ^b^
**Nationwide**	**8**	**3%**
**Dublin**	**43**	**16.2%**
**Mid-east**	**31**	**11.7%**
Kildare	10	3.8%
Louth	7	2.6%
Meath	7	2.6%
Wicklow	7	2.6%
**South-east**	**37**	**14.0%**
Carlow	7	2.6%
Kilkenny	7	2.6%
Waterford	15	5.7%
Wexford	8	3.0%
**South-west**	**29**	**10.9%**
Cork	22	8.3%
Kerry	7	2.6%
**Mid-west**	**21**	**7.9%**
Clare	7	2.6%
Limerick	7	2.6%
Tipperary	7	2.6%
**Midland**	**28**	**10.5%**
Laois	7	2.6%
Longford	7	2.6%
Offaly	7	2.6%
Westmeath	7	2.6%
**Border**	**36**	**13.5%**
Cavan	8	3.0%
Donegal	7	2.6%
Leitrim	7	2.6%
Monaghan	7	2.6%
Sligo	7	2.6%
**West**	**29**	**10.9%**
Galway	15	5.7%
Mayo	7	2.6%
Roscommon	7	2.6%

^a^Region In order of population density from lowest to highest

^b^Location not provided for three services

The most commonly provided services were physiotherapy (12%) and occupational therapy (11%) ( Table 3). Informants stated that 243 services (92%) were delivered through public healthcare and 23 (9%) were provided through private healthcare. This information was unknown for 11 services (4%). A website was provided for 240 services (91%). However, in many cases the website provided related to the organisation rather than the specific service provide for people with CP within the organisation. Informants stated that they or someone they knew (aged 18 years and over) received assessment, treatment, or support for their CP at 259 services (98%). For 196 services (74%), informants did not respond to this question or stated they did not know if the service had expertise in treating or supporting adults with CP.

**Table 3.** Service type (n=265).

Service type	Number of unique services	%
Physiotherapy	33	12.5
Occupational therapy	30	11.3
Home help/personal care	29	10.9
Personal assistance ^a^	29	10.9
Speech and language therapy	28	10.6
Social work	27	10.2
Psychiatry/counselling	27	10.2
Day service	9	3.4
Assistive Technology and Special Seating (ATSS)	8	3.0
Hydrotherapy	5	1.9
Training and employment service	4	1.5
Adult service/service for adults with physical disabilities ^b^	4	1.5
Assisted/independent living	4	1.5
Advocacy service	3	1.1
Orthotics and prosthetics	3	1.1
Sports group or clubs	3	1.1
Strengthening and exercise facilities	3	1.1
Respite	3	1.1
Relaxation, meditation and massage therapy	2	0.8
Medical neuro team	1	0.8
Neurology	1	0.4
Pain clinic	1	0.4
Wheelchair accessible beaches	1	0.4
Resource centre	1	0.4
Social club	1	0.4
Driving school	1	0.4
Feeding eating and drinking	1	0.4
Hand function	1	0.4
Orthopaedics	1	0.4
Residential house	1	0.4

^a^during verification, we identified that adults with CP were not eligible for one of these services

^**b**^One service listed as “adult service” by a respondent did not exist. Instead, the organisation provided information about the following services separately: physiotherapy, occupational therapy, speech and language therapy, hydrotherapy, psychology, social work, dietetics, positive behavioural therapy, residential & day service, and music/art/leisure.

### Data checking

Table 4 reports the number of services for which we obtained information online and from organisations. Of the 265 services provided by informants, we confirmed the organisation name against online information for 263 services (99.3%). We confirmed the service name for 89 services (34%) against online information. Where we were unable to find the service name online, we were unable to verify any subsequent information for the service. One informant provided information about 180 services provided by HSE “disability services” (e.g. occupational therapy provided by HSE disability services in Meath). Of these, we identified online information about 20 services (11.1%) that were specifically provided as part of “disability services”. For an additional 69 services (38%), we identified information about a general service provided by the HSE but could not determine if it was part of the disability service. We did not count these 69 services as being verified online.

**Table 4.** Number of services for which the following information was obtained online or from the organisation.

	Data available online, n (%) ^a^	Data provided by organisation, n (%) ^a^
Organisation name	263 (99.3%)	27 (10.2%)
Service name	89 (33.6%)	26 (9.8%)
Website or webpage for the service	87 (32.8%)	27 (10.2%)
Service location	84 (31.7%)	27 (10.2%)
Specialist CP service	5 (1.9%)	26 (9.8%)
**Eligibility criteria**		
Lower age boundary	26 (9.8%)	26 (9.8%)
Upper age boundary	22 (8.3%)	26 (9.8%)
Available to people without intellectual disability	70 (26.4%)	25 (9.4%)
Other criteria	26 (9.8%)	26 (9.8%)
**Type of supports provided**		
Transitional support	0	26 (9.8%)
MDT review	6 (2.3%)	24 (9.0%)
Annual review	0	24 (9.0%)
Review by a professional with expertise in vocational skills	0	24 (9.0%)
Referral to a professional with expertise in vocational skills	0	24 (9.0%)
Review by a professional with expertise in independent living	0	24 (9.0%)
Referral to a professional with expertise in independent living	0	26 (9.8%)
Speech and language therapy assessment	8 (3.0%)	25 (9.4%)

^a^calculated as a percentage of total number of services (i.e., 265)

We were unable to find contact details for 169 services. For the remaining services, we asked organisations to complete a survey to provide details about their service(s). We received data from eight organisations about 27 services ( Table 4). Of the 265 services provided by informants in the original survey, we received information from the organisation to verify organisation name for 27 services (10%), service name for 25 services (9%), service location for 26 services (10%), service type for 25 services (9%), and a website for 19 services (7%). We received data on eligibility criteria and types of supports provided for 25 or 26 services (9% or 10%). In two cases, the service name provided by the organisation did not match the service name provided by the informant in the original survey. Furthermore, the website provided by the organisation did not always relate to the specific service.

Where data about 27 services were provided by organisations, 21 services were available to adults with CP without intellectual disability. The lower age boundary was 18 years for 22 services, 11 years for one service, and three services had no lower age criteria. There was no upper age boundary for 23 services. The upper age boundary was 30 years for one service, 65 years for one service, and one service was currently reviewing their upper age criteria. Twenty-four services had expert knowledge and/or skills in supporting adults with CP.

Organisations stated if each service provided the following: transitional support (n=10); review by a specialist multidisciplinary team (n=13); an annual review with a healthcare professional with expertise in neurodisability (n=11); a review by a professional with expertise in vocational skills (n=5); review by a professional with expertise in independent living (n=5); referral to a professional with expertise in vocational skills (n=6); referral to a professional with expertise in independent living (n=7); assessment by speech and language therapy services (n=5). However, in the free text box, organisations reported there were constraints as to who could access these supports because of limited resources.

### Feedback

Feedback was provided by three informants and included: (1) providing a list of services for informants to add to, rather than asking them to identify services, to prevent duplication between respondents; (2) including medical consultant as a role descriptor and an example of a health service when asking for service details; (3) listing “CP sports” as an example of a health service.

## Discussion

This study aimed to pilot and refine a methodology to map services for adults with CP in Ireland. Adults with CP, families and service providers identified 265 unique services in 30 different organisations. Services fell under 23 broad types of service, with physiotherapy and occupational therapy the most commonly reported. However, we encountered challenges checking the data provided by informants. We could only find 34% of services online. Basic information about eligibility criteria for accessing these services such as age criteria was available online for less than 30% of services. For nearly all services, it was impossible to determine from online information if they provided the supports outlined in the NICE Quality Standard for adults with CP ^21^. Our findings highlight the challenges that adults with CP, families and health professionals face when attempting to identify health services for adults with CP. 

We chose to use a multi-informant mapping methodology because it was developed for a recent study as an efficient way to collate data on health services for adults with ADHD in the United Kingdom ^17, 18^. However, we deemed it necessary to pilot the methodology to ensure we collected accurate data. Indeed, we identified several challenges to collecting data on services for adults with CP, emphasising the importance of this piloting phase. The key challenges can be summarised as those relating to: 1) the scope of the service map; 2) reliability of information provided by informants; and 3) verifying the information provided by informants.

Adults with CP want information about services that support the transition to adulthood, beyond health services, such as those relating to education, advocacy, independent living and employment ^13^. However, we recognised that creating a map of services relating to all of these areas would substantially increase the quantity of data obtained and the resources needed to successfully complete a study. In partnership with our PPI contributors, we chose to focus on health services. We used a broad definition of health services so that the data collected were useful to adults and families. Despite using a broad definition and providing examples of health services, we still received data on services that did not meet our definition, or there was ambiguity as to whether they met the definition. When this methodology was previously used to collect data on services for adults with ADHD population, similar challenges in deciding the scope of services were identified ^17^. Prior to conducting a larger study, it may be necessary to narrow the scope of services included, and develop and adhere to strict inclusion criteria. Although narrowing the scope of a future study will make it more feasible to collect accurate data, it will exclude data on services that are vitally needed by adults with CP and families. It is therefore important to consider an appropriate balance of feasibility and impact. Ultimately, however, from our experience it will be necessary to compromise on the range of services included in a service map in order to complete the project and provide accurate data.

The methodology used relied on informants to provide data on services. Although we identified additional services online that had not been provided by informants when checking data, we did not report these. We were able to verify 99% of the organisations provided by informants against online information, suggesting that informants provide accurate data on organisation names. However, the data that informants provided about services appeared to be less accurate. We received responses from informants (service users and service providers) that highly likely related to the same service in an organisation, but stated different service names. For example, two informants may have referred to the same service as “adult services” and “physiotherapy”. Further, the service name provided by informants may not be the same as that used by the organisation. This may explain why we were only able to identify 34% of services online. For example, an informant provided data on one “adult service” in an organisation, while the organisation provided data on ten specific services for adults. Additionally, 205 of the 265 services identified were reported by one informant. This informant reported 180 “disability services” provided by the HSE across Ireland. We were unable to find information about the majority of these services online, which strongly influenced our results regarding data verification. However, based on the methodology we used, we had no justification for excluding data from this individual.

Finally, verifying data provided by informants was challenging. This was partly because the data provided by informants did not match the data provided online. However, there was also a lack of information about services online. Although a website was provided for 89% of the services, they were mostly websites for the organisation rather than the specific service. Many organisations did not state online the services they provided to adults with CP. When the service was listed online, eligibility criteria for access was rarely provided. Our attempts to verify information online demonstrates how difficult it can be for adults with CP and their families to firstly identify services and, secondly, determine if they are eligible to attend them. International literature similarly reports that adults with CP have challenges navigating services and obtaining information ^9^. The challenges with finding accurate information online demonstrates that it is essential to obtain information directly from organisations. However, we had a very low response rate from our direct requests to organisations. This meant the majority of information about services provided by informants was not verified by organisations, so the findings should be interpreted with caution.

Future service mapping studies should ensure that a list of pre-identified services be included for informants. The data collected in this study can be used to compile such a list. Indeed, this approach was taken in a study to map services for adults with ADHD after an initial pilot study was conducted ^17^, and was suggested by an informant in the current study. Further it may be necessary to send survey questions to organisations via Freedom of Information requests (right to request information from public sector organisation) to obtain data as this approach was shown to increase response rate ^16^. Opening the survey to informants for a longer period may also increase response rate.

Adults with CP, families and health professionals consistently report wanting a ‘map’ of services to make it easier for them to navigate adult health and social care services. Adults with CP on the research team believe it is important to share the following information about services: address; contact information; website; whether or not it is verified that an adult with CP has used the service; whether or not informants considered the service had expertise in supporting adults with CP; if the service is available through the public or private health system; and, eligibility criteria to access the service. However, given the challenges we had when verifying service information, it will be difficult to ensure accuracy of submitted service details. Furthermore, if a map of services is created, a long-term plan for updating, curating and disseminating the information needs to be developed. Despite concerns about providing accurate data on services, adults with CP on the research team believe we should still share a map of the services we identified, with the caveat that the map is incomplete and that it was not possible to verify the majority of the information.

## Conclusion

To conclude, this study highlighted the complexity of mapping services for adults with CP in Ireland. We were unable to ensure that the majority of data collected on health services for adults with CP in this study was accurate. We therefore recommend that an alternate methodology is identified or developed to map services for adults with CP in Ireland.

## Data Availability

Zenodo: Mapping health services for adults with cerebral palsy in Ireland: a pilot study https://doi.org/10.5281/zenodo.7051708 ^22^ This project contains the following underlying data: Metadata.docx service_data.xls Zenodo: Mapping health services for adults with cerebral palsy in Ireland: a pilot study https://doi.org/10.5281/zenodo.7051708 ^22^ This project contains the following extended data: Survey copy.pdf Data are available under the terms of the Creative Commons Attribution 4.0 International license (CC-BY 4.0).
