# Supplementary material for: Mapping health services for adults with cerebral palsy in Ireland: a pilot study
Source: HRB Open Res. 2024 Feb 8;5:61. Originally published 2022 Sep 20. [Version 3] doi: 10.12688/hrbopenres.13609.3 (PMC10603314; doi:10.12688/hrbopenres.13609.3)
Supplement: Peer review history

# Reviewer report: Reviewer response for version 3

**DOI:** 10.21956/hrbopenres.15205.r38096

**Published:** 2024-02-20

**Authors:** Prue Morgan

**Affiliations:** 1 Department of Physiotherapy, School of Primary and Allied Health Care, Monash University, Melbourne, Vic, Australia

Amendments have been made to my satisfaction.

 No further comments to make.

 This version is approved.

Is the work clearly and accurately presented and does it cite the current literature?

Yes

If applicable, is the statistical analysis and its interpretation appropriate?

Yes

Are all the source data underlying the results available to ensure full reproducibility?

No source data required

Is the study design appropriate and is the work technically sound?

Yes

Are the conclusions drawn adequately supported by the results?

Partly

Are sufficient details of methods and analysis provided to allow replication by others?

Partly

Reviewer Expertise:

Lifespan care of adults with cerebral palsy

I confirm that I have read this submission and believe that I have an appropriate level of expertise to confirm that it is of an acceptable scientific standard.

# Reviewer report: Reviewer response for version 2

**DOI:** 10.21956/hrbopenres.14943.r36570

**Published:** 2023-10-31

**Authors:** Marco Konings

**Affiliations:** 1 KU Leuven, Leuven, Belgium

The transition period from children’s to adult’s services is known to be particularly challenging for people with CP because they no longer have access to coordinated care and have difficulty identifying and navigating services to meet their needs. The authors of this study aimed to pilot and refine a multi-informant mapping methodology to map services for adults with CP in Ireland.

 INTRODUCTION

- Limited information is provided on the rationale for choosing this specific multi-informant mapping methodology (that was used previously to efficiently collate and communicate data on health services for adults with ADHD in the United Kingdom as stated by the authors). More information on this decision for this methodology would be helpful for the interpretation of the results.

METHODS

*Informants*

- Informants were identified in their Methods section through the research team’s network. Could the authors also please comment on a possible selection bias within the identified informants, and its possible impact on the results?
- Why did the authors decide to include also service providers working in paediatric settings as informants? (instead of using only service providers working in adult setting?

*Data collection*

- The authors state that because this study was to pilot and refine the methodology, they limited the number of people who received the survey so that they could refine the methodology based on their responses before distributing it more widely. Could the authors please comment (e.g. in the Discussion section) if this decision to limit the number of people who received the survey may have impacted the effectiveness of the piloted methodology (or why not)?
- Throughout the manuscript the authors refer several times to the “the survey”. However, if I understand it correctly, two types of surveys have been used in this study: one addressed to the informants, and to the identified organisations? Could the authors throughout the manuscript state explicitly if they are talking about the ‘informants survey’, or about the ‘identified organizations survey’ at each reference to a survey in the manuscript?
- The opportunity to provide anonymous feedback on the informants survey seemed to have been used only very minimally by the informants. Did the authors considered other approaches to receive feedback on the used survey (which is quite essential in the used methodology), or have any advices on how this could be done better in future research?

*Data checking *

- The authors checked data for each unique service by (1) searching online and (2) sending a survey to the providing organisation. Could the authors please specify in text if these two approaches were performed in parallel or sequentially?

RESULTS

- Could the authors specify throughout the manuscript and for all tables if the presented results involve 1) organisations and/or services as described by the informants (unverified), or 2) organisations and/or services that have been verified. This has been a bit unclear to me sometimes.

*Description of informants*

- Only 24 out of 39 approached informants completed the survey. Could the authors please comment on the possible impact of this response rate on their results?
- The authors state “ Service providers stated they worked in the voluntary sector only, HSE only, private sector only, or HSE and voluntary sector.” Could they please add here how many per category (n=?)?

 
*Description of services*

- The authors state in the manuscript: “in Eight services (3%) were described as nationwide. Forty-three services (16%) were located in Dublin, 37 (14%) in the south-east region, and 36% (13%) in the border region.” As this seems repetition of the data reported in Table 2, it could be removed as text in the manuscript.
- Am I correct that the listed ‘types or services provided’ in Table 1 are the data from informants prior to the verification process? If yes, would it be possible to provide an overview table of all verified services and organisations?

DISCUSSION

- The authors identify three key challenges as those relating to: 1) the scope of the service map; 2) reliability of information provided by informants; and 3) verifying the information provided by informants.
  It is a bit unclear to me what is meant exactly with “the scope of the service map”? Could the authors please clarify?
  In addition, could the authors please explain based on what they have come to these three challenges? That is, both challenges 2) and 3) seem fair possibilities and either one could be true, however, I am not sure it can be concluded from the results of this study? I would tend to agree with the authors it is likely a mix of both, but I think it cannot be excluded based on the presented data that for example the informants were completely reliable but the verification process was not good enough, or vice versa?

- Could the authors please comment on why they think the response rate from the requests to the organisations was very low?
- One informant identified 205 of 265 services, however I understand the notion that based on the methodology you used, there was no justification to exclude the data of this individual. However, in line with the study aim to refine the methodology, would the authors have any suggestion or idea on how to deal with or prevent this in future studies?
- The authors remark that “Prior to conducting a larger study, it may be necessary to narrow the scope of services included”. Did the authors also consider the options to narrow the geographical range of the study? E.g. county-wide instead of nation-wide to make it more feasible?

CONCLUSION

- The conclusion of the authors is a bit vague and it is unclear to me why the authors recommend an ‘alternate’ methodology (but without specifying possible alternatives) rather than an adapted or refined methodology?
  In this respect, would it be possible to move to the Conclusion this part of the Discussion:
  “Prior to conducting a larger study, it may be necessary to narrow the scope of services included, and develop and adhere to strict inclusion criteria. Although narrowing the scope of a future study will make it more feasible to collect accurate data, it will exclude data on services that are vitally needed by adults with CP and families. It is therefore important to consider an appropriate balance of feasibility and impact. Ultimately, however, from our experience it will be necessary to compromise on the range of services included in a service map in order to complete the project and provide accurate data.“?

Is the work clearly and accurately presented and does it cite the current literature?

Yes

If applicable, is the statistical analysis and its interpretation appropriate?

Yes

Are all the source data underlying the results available to ensure full reproducibility?

Yes

Is the study design appropriate and is the work technically sound?

Yes

Are the conclusions drawn adequately supported by the results?

Partly

Are sufficient details of methods and analysis provided to allow replication by others?

Yes

Reviewer Expertise:

Cerebral palsy

I confirm that I have read this submission and believe that I have an appropriate level of expertise to confirm that it is of an acceptable scientific standard, however I have significant reservations, as outlined above.

# Reviewer report: Reviewer response for version 2

**DOI:** 10.21956/hrbopenres.14943.r35408

**Published:** 2023-10-26

**Authors:** Meghan E. Munger

**Affiliations:** 1 Gillette Children's Specialty Healthcare, Saint Paul, Minnesota, USA

Overall: The purpose was to pilot methodology to map services for adults living with CP in Ireland. In order to make this more generalizable, it would have been interesting to approach the problem as bigger than affecting just adult CP in Ireland - rather, a methodology that anyone with a similar problem could use to apply. As written, it is framed as a challenge unique to Ireland. This isn't inherently a problem, but may be more interesting to more readers if approached more broadly and then simply applied to Ireland as the study sample.

 Intro: Your entire paragraph first paragraph could likely be trimmed to a single sentence or two to get to your point sooner. I think the bigger point is that CP is complex and childhood-onset, these complex children transition to adulthood where things get more complex, but there is a gap in understanding of services available to these adults.

 Methods: It's great you partnered with those with lived experiences. It isn't clear that you had any service providers that treated adults with CP? You conclude that your methodology is not sufficient, but you had a very short window of time for data collection so it isn't clear whether the method is the problem or your length of study. It's unclear why you treated "nationwide" and "countywide" services differently. You give examples of what health services could be included with your definition, but not examples of what you excluded. That would be helpful.

 Discussion: A huge finding that could be highlighted even more is how challenging it is for adults or their caregivers to navigate available health services online. Clearly insufficient information is provided. Agree that open-ended text responses provides challenges from a data checking perspective. I'm not sure the value of reporting your methods differently from your final product. Overall, this feels more like a quality improvement type study than a research study.

Is the work clearly and accurately presented and does it cite the current literature?

Partly

If applicable, is the statistical analysis and its interpretation appropriate?

Not applicable

Are all the source data underlying the results available to ensure full reproducibility?

No source data required

Is the study design appropriate and is the work technically sound?

Partly

Are the conclusions drawn adequately supported by the results?

Partly

Are sufficient details of methods and analysis provided to allow replication by others?

Yes

Reviewer Expertise:

children with medical complexity, health services research

I confirm that I have read this submission and believe that I have an appropriate level of expertise to confirm that it is of an acceptable scientific standard, however I have significant reservations, as outlined above.

# Reviewer report: Reviewer response for version 2

**DOI:** 10.21956/hrbopenres.14943.r33268

**Published:** 2022-12-19

**Authors:** Prue Morgan

**Affiliations:** 1 Department of Physiotherapy, School of Primary and Allied Health Care, Monash University, Melbourne, Vic, Australia

I have reviewed the updated version of the manuscript and am now satisfied that it has addressed concerns raised in the previous version.

 I have no further comments to make.

Is the work clearly and accurately presented and does it cite the current literature?

Yes

If applicable, is the statistical analysis and its interpretation appropriate?

Yes

Are all the source data underlying the results available to ensure full reproducibility?

No source data required

Is the study design appropriate and is the work technically sound?

Yes

Are the conclusions drawn adequately supported by the results?

Partly

Are sufficient details of methods and analysis provided to allow replication by others?

Partly

Reviewer Expertise:

Lifespan care of adults with cerebral palsy

I confirm that I have read this submission and believe that I have an appropriate level of expertise to confirm that it is of an acceptable scientific standard.

# Reviewer report: Reviewer response for version 1

**DOI:** 10.21956/hrbopenres.14878.r32891

**Published:** 2022-10-21

**Authors:** Elisabet Rodby-Bousquet

**Affiliations:** 1 Department of Clinical Sciences, Orthopaedics, Lund University, Lund, Sweden

Access to health care for adults with cerebral palsy (CP) is challenging and often described as fragmented with a lack of expertise and coordination of care. The aim of this study was to pilot and refine a methodology to map services for adults with CP in Ireland. They used a multi-informant mapping methodology. The authors highlight the complexity of mapping services for adults with CP, e.g. reliability of information and difficulties verifying the information. In addition, they suggest improvements for future research. I'd like to commend the authors for their patient involvement throughout the research process.

**Author Details:**

 There is a small typing error for row number 4: " ^4 ^4. Public and Patient Involvement contributor."

**Introduction:**

 The introduction provides a good overview of the topic and a clear rationale for the study.

**Methods and Results:**

 I acknowledge the complexity of gathering information about different services. However, mapping organisations by their "company" names doesn't provide much information about their type of services. I suggest you group them by type of service with additional information about if it's specialised health care, primary health care, charity, private or other organisations such as sports centres (swimming pools, gyms). This would make it easier to interpret the findings also for readers outside Ireland.

 The time slot for reporting the survey was just a bit over a month and during the summer period (August). Potentially, this could have influenced the low number of respondents.

 Considering the low number of service providers (n=9), I think the reporting of numbers is sufficient and that percentages do not provide any additional information, e.g. two were paediatricians (22%) and one was a social worker (11%).

**Discussion:**

 The complexity of mapping and verifying the accuracy of services for adults with CP are highlighted. Only a small proportion of the services could be verified. So, the findings should be interpreted with caution.

Is the work clearly and accurately presented and does it cite the current literature?

Yes

If applicable, is the statistical analysis and its interpretation appropriate?

Yes

Are all the source data underlying the results available to ensure full reproducibility?

Yes

Is the study design appropriate and is the work technically sound?

Yes

Are the conclusions drawn adequately supported by the results?

Yes

Are sufficient details of methods and analysis provided to allow replication by others?

Yes

Reviewer Expertise:

Cerebral palsy

I confirm that I have read this submission and believe that I have an appropriate level of expertise to confirm that it is of an acceptable scientific standard, however I have significant reservations, as outlined above.

# Response

**Published:** 2022-12-08

**Authors:** Manjula Manikandan

**Affiliations:** Royal College of Surgeons in Ireland, Ireland

**Author Details: **The manuscript draft is updated.

**Methods: **Thank you for this suggestion. We agree that mapping organisations by name does not provide much information about the type of services and lacks sufficient detail to be useful, particularly to people outside of Ireland.

 However, it is challenging to categorise the organisations by type of service because some organisations provide multiple services. We also don’t have data on whether the service is specialised healthcare, primary health care etc. We collected data on whether the service was provided through public or private healthcare. We have included the type of service(s) provided by the organisation and whether the organisation provided public or private healthcare to Table 1.   

 Yes, the survey was open to informants between 28 ^th^ July and 3 ^rd^ September. This could have potentially reduced the number of respondents when the initial survey was open. However, data checking and verification continued beyond the summer period and this does not necessarily explain the low response from organisations.

 We have added to the discussion that opening the survey to respondents for a longer period may also increase response rate.

**Results:**

 We updated the manuscript as suggested below:

 “Of the nine service providers, three were physiotherapists, three were occupational therapists, two were paediatricians and one was a social worker.”

**Discussion:**

 Yes, we have addressed this to the discussion section :

 “However, we had a very low response rate from our direct requests to organisations. This meant the majority of information about services provided by informants was not verified by organisations, so the findings should be interpreted with caution.”

 We have also amended our conclusion to read:

 “To conclude, this study highlighted the complexity of mapping services for adults with CP in Ireland. We were unable to ensure that the majority of data collected on health services for adults with CP in this study was accurate. We therefore recommend that an alternate methodology is identified or developed to map services for adults with CP in Ireland.”

# Reviewer report: Reviewer response for version 1

**DOI:** 10.21956/hrbopenres.14878.r32894

**Published:** 2022-10-05

**Authors:** Prue Morgan

**Affiliations:** 1 Department of Physiotherapy, School of Primary and Allied Health Care, Monash University, Melbourne, Vic, Australia

Identifying and enhancing services for lifespan care of people with cerebral palsy has been a growing area of focus for clinicians and researchers. This study aimed to map health services for adults with CP in Ireland.

**Introduction/Method:**

 An overview of the need for the study, and prior available information is provided - however, it would be helpful for international readers to include the total number of people with CP currently living in Ireland to enable potential comparisons with other countries.

 The multi informant mapping methodology previously used to identify services for ADHD in UK is referenced, although it is unclear (without going to the primary reference) whether the identical methodology was used or only components. 

 My main concern relates to the health services definition and inclusion of exercise groups, gyms or swimming pools. I suspect this expanded definition may lack clarity regarding suitability or otherwise for adults with CP and starts to dilute the specificity of the data regarding health services. I would like some assurances from the authors regarding the veracity of this definition.

**Results:**

 As this was a pilot study, the data provided is relatively unrefined in terms of service descriptions and I was unable to interpret much of the Ireland-specific geography eg Table 2. 

 Data checking - perhaps not surprisingly, much of the data provided by survey respondents was unable to be verified by the authors, making interpretation and conclusions difficult other than 'we don't know' e.g. very low % of service verification (34%). I think the results, therefore, are over-reported given the low rate of verified data.

**Discussion:**

 A reasonable overview of the significant limitations of the survey methodology is provided here - in particular, the challenge of identifying scope of services, information reliability and verification. I don't think that any strong conclusions, however, can be drawn from the limited data sourced.

 Beyond this conclusion regarding the significant limitations of the methodology, I don't believe the data provided by this pilot study supports further exploration other than indicating the difficulty of the mapping process and I would support an alternate methodology in the future.

Is the work clearly and accurately presented and does it cite the current literature?

Yes

If applicable, is the statistical analysis and its interpretation appropriate?

Yes

Are all the source data underlying the results available to ensure full reproducibility?

No source data required

Is the study design appropriate and is the work technically sound?

Yes

Are the conclusions drawn adequately supported by the results?

Partly

Are sufficient details of methods and analysis provided to allow replication by others?

Partly

Reviewer Expertise:

Lifespan care of adults with cerebral palsy

I confirm that I have read this submission and believe that I have an appropriate level of expertise to confirm that it is of an acceptable scientific standard, however I have significant reservations, as outlined above.

# Response

**Published:** 2022-12-08

**Authors:** Manjula Manikandan

**Affiliations:** Royal College of Surgeons in Ireland, Ireland

**Introduction:**

 There is no national CP register in the republic of Ireland, therefore we estimated the prevalence of CP in adults from the Northern Ireland Cerebral Palsy register report published recently, which was 2.38 per 1000 population, born between 1981-2001 (McConnell et al 2021).

 Based on the prevalence of CP among adults in Northern Ireland (McConnell et al 2021) and the population of adults in the republic of Ireland (Central statistics office, 2017) we estimate that there are approximately 6,800 adults aged 19-64 years living with CP in Ireland.

 We updated the manuscript introduction as follows:

 "The prevalence of CP among adults reported recently from the Northern Ireland CP Register (NICPR) was 2.38 per 1000 population born between 1981-2001. ^7^ Based on the NICPR report ^7^ and the population of adults in the Republic of Ireland ^5^ we estimate there are approximately 6,800 adults living with CP in Ireland." 

**Method:**

 In this pilot study we used 5 of the 7 steps of the multi-informant identical methodology that was previously used to map services for ADHD in the UK (Price et al 2019, 2020). This was described in the methods section below:

 “We piloted a methodology used to map services for adults with ADHD in the UK.

 The methodology consisted of seven steps: 1. Defining health services; 2. Identifying informants; 3. Designing a survey; 4. Collecting data; 5. Data checking and analysis; 6. Communicating findings; and 7. Updating findings ^17^.”

 “We report data relating to steps 1-5 and share plans for communicating findings (i.e., step 6).”

 The research team includes adults with CP who contributed to all stages of this study and are co-authors of this paper.

 We extensively discussed the definition of health services, and what services should be included and excluded, with these contributors. As described in the methods, we defined health services, based on the definition by Nick and Reinhold 2005, as

  “ *those that are first and foremost undertaken to have direct effect on people’s health. These extend from **health promotion** and disease prevention, through curative services, to long-term care and rehabilitation”.*

 The adults with CP who are part of our research team argued that exercise groups, gyms and swimming pools support health promotion and it is important for adults with CP to have information about this, because they can be difficult to find. Thus, as described in the methods, “we also included exercise groups, gyms or swimming pools that are accessible to people with disability in the examples of services because they support health promotion and adults with CP on the research team thought information on these facilities is important to collate”.  

 However, we acknowledge in the discussion that we included a broad definition of health services and that “it may be necessary to narrow the scope of services included and develop and adhere to strict inclusion criteria.” This may include excluded exercise groups, gyms and swimming pools because of difficulty collecting data about these facilities. However, given the adults with CP on our team thought it was very important to include these, removal of these from any future study that attempts to map health services would require discussion and compromise with adults with CP.

**Results:**

 We have included the type of service(s) provided by the organisation and whether the organisation provided public or private healthcare to Table 1.  

 In Table 2, we have categorized the counties into regions according to population density (listed from highest density to lowest density).

**Data checking:**

 As we were unable to verify the majority of services provided by informants, we limit are description of the services provided by informants to:

1. Name of organisation providing the service
2. County in which the service was located
3. If the service was delivered through public or private healthcare.
4. Type of service provided
5. If a website was provided by the key informant.
6. If the informant or someone they knew received assessment, treatment or support for their CP
7. If the service had expertise in treating or supporting adults with CP.

We have removed the data relating to point 7 (i.e. if the service had expertise in treating or supporting adults with CP) from the methods and results, in order to reduce reporting of the results.

 We would appreciate further clarity about how the results are over-reported and are happy to make further changes to address this. 

 We describe the challenges with verifying data in the discussion (as outlined below).

Discussion

 “Finally, verifying data provided by informants was challenging. This was partly because the data provided by informants did not match the data provided online. However, there was also a lack of information about services online. Although a website was provided for 89% of the services, they were mostly websites for the organisation rather than the specific service. Many organisations did not state online the services they provided to adults with CP. When the service was listed online, eligibility criteria for access was rarely provided. Our attempts to verify information online demonstrates how difficult it can be for adults with CP and their families to firstly identify services and, secondly, determine if they are eligible to attend them. International literature similarly reports that adults with CP have challenges navigating services and obtaining information. ^9^ The challenges with finding accurate information online demonstrates that it is essential to obtain information directly from organisations. However, we had a very low response rate from our direct requests to organisations. This meant the majority of information about services provided by informants was not verified by organisations.”

**Discussion:**

 We agree that a strong conclusion cannot be drawn from this pilot study and that an alternate method of mapping services for adults with CP should be identified or developed to use in the future.

 We have removed some information from the discussion about the services provided by adults with CP, because they are questionable due to the limited data source.

 Specifically, we removed the following sentences:

 When this information was provided by an organisation, only between 5 and 14 services provided a support outlined in the Quality Standard. Even when provided, some organisations stipulated limits as to who could avail of these supports.

 The findings also potentially indicate that few services offer supports that NICE consider to be ‘high-quality care in priority areas for improvement’ for adults with CP.

 We updated the conclusion as follows:

Conclusion

 “To conclude, this study highlighted the complexity of mapping services for adults with CP in Ireland. We were unable to ensure that the majority of data collected on health services for adults with CP in this study was accurate. We therefore recommend that an alternate methodology is identified or developed to map services for adults with CP in Ireland.”

Abstract

**“**Conclusion: This pilot study highlighted the complexity of mapping services for adults with CP in Ireland. We recommend that an alternative methodology should be used to map services for adults with CP in Ireland.”
